# Systemic Inflammatory Indices and Disease Severity in Acute Colonic Pseudo-Obstruction: An Exploratory Retrospective Study

**DOI:** 10.3390/diagnostics16040601

**Published:** 2026-02-18

**Authors:** Çağrı Akalın, Mümin Demir, Gökhan Zaim

**Affiliations:** Department of General Surgery, Faculty of Medicine, Ordu University, Ordu 52200, Türkiye

**Keywords:** acute colonic pseudo-obstruction, Ogilvie’s syndrome, systemic inflammation response index, systemic immune-inflammation index, neutrophil-to-lymphocyte ratio, biomarkers, surgical intervention

## Abstract

**Background**: Acute colonic pseudo-obstruction (ACPO) is associated with substantial morbidity and mortality, particularly when complicated by ischemia or perforation. Although radiological assessment remains central to clinical monitoring, objective biomarkers reflecting disease severity and clinical course are limited. This study was designed as an exploratory, hypothesis-generating analysis to examine associations between composite systemic inflammatory indices and ACPO severity. **Methods**: In this retrospective observational study, 47 patients diagnosed with ACPO and 50 age- and sex-matched healthy controls were analyzed. Neutrophil-to-lymphocyte ratio (NLR), systemic immune-inflammation index (SII), and systemic inflammation response index (SIRI) were calculated from peripheral blood counts obtained at hospital admission. Associations between these indices and subsequent surgical intervention were evaluated, recognizing surgery as a decision-dependent clinical endpoint. **Results**: SIRI and SII values were significantly higher in patients with ACPO compared with controls. Within the ACPO cohort, NLR and SIRI demonstrated the strongest within-cohort discrimination for surgical intervention (AUC 0.840, 95% CI: 0.722–0.958 and AUC 0.835, 95% CI: 0.718–0.952, respectively). A multivariate model incorporating SIRI (>3.52) and colonic diameter (>12 cm) achieved 88.9% within-sample classification accuracy. **Conclusions**: This exploratory study demonstrates that composite inflammatory indices derived from routine blood counts are associated with disease severity and clinical course in ACPO. These preliminary findings require validation through larger, prospective, multicenter studies incorporating disease control groups, objective outcome measures, and formal validation frameworks before any clinical utility can be established.

## 1. Introduction

Acute colonic pseudo-obstruction (ACPO), commonly referred to as Ogilvie’s syndrome, is a rare but potentially life-threatening clinical entity characterized by massive colonic distension in the absence of an intrinsic or extrinsic mechanical obstructing lesion [1,2]. This condition typically manifests in elderly, debilitated patients with multiple comorbidities or those who have recently undergone major non-abdominal surgery, such as orthopedic or spinal procedures [3,4]. Although its exact pathogenesis remains incompletely understood, it is widely accepted that ACPO results from autonomic dysregulation—specifically involving a disruption of the excitatory parasympathetic supply to the distal colon or a relative excess of inhibitory sympathetic tone [5,6]. This dysautonomia leads to impaired colonic motility, vascular congestion, and progressive distension, initiating an ischemic cascade and potential hemodynamic compromise.

Early recognition and timely intervention are critical for patient survival, as massive colonic dilatation can impair intramural blood flow, resulting in ischemia, gangrene, and eventual visceral perforation [7]. While perforation occurs in only 1–3% of cases, mortality among patients with perforation or ischemia is substantial, ranging from 35% to 72% [2,8]. Management currently relies heavily on serial radiological monitoring of colonic diameter. However, radiological findings often exhibit a significant time lag relative to the biological onset of ischemia and are subject to substantial inter-observer variability. This diagnostic challenge is particularly pronounced in elderly or sedated patients, where physical examination findings are frequently masked and clinical symptoms are unreliable, often leading to delayed recognition of vascular compromise [8].

While radiological measurement of colonic diameter remains the diagnostic cornerstone, it lacks the prognostic precision required to identify patients at high risk for clinical deterioration. Consequently, identifying hematologic indices that provide an early, systemic reflection of impending ischemia could potentially inform future approaches to ACPO management. Recently, novel inflammatory biomarkers—specifically the neutrophil-to-lymphocyte ratio (NLR), the systemic immune-inflammation index (SII), and the systemic inflammation response index (SIRI)—have emerged as potent, cost-effective tools for monitoring systemic inflammatory responses and predicting outcomes in various gastrointestinal emergencies. These composite indices have demonstrated associations with disease severity in diverticulitis, acute appendicitis, cholecystitis, strangulated inguinal hernias, benign intestinal obstructions, and adhesive small bowel obstructions [9,10,11,12,13,14]. Unlike isolated cell counts, these composite indices provide an integrated assessment of the host’s immune-inflammatory status by simultaneously capturing neutrophilia, lymphopenia, and monocytosis or thrombocytosis [15,16].

Despite their reported associations with outcomes in other acute surgical conditions, the prognostic value of NLR, SII, and SIRI in ACPO remains unexplored. This study evaluated the relationship between these hematologic inflammatory indices and outcomes in ACPO, with particular focus on their association with subsequent surgical intervention.

## 2. Materials and Methods

### 2.1. Study Design and Ethical Considerations

This retrospective comparative study was conducted at the Ordu University Training and Research Hospital, Department of General Surgery, covering the period between January 2014 and August 2025. The study protocol was reviewed and approved by the Ordu University Non-Interventional Clinical Research Ethics Committee (Decision No: 290; Date: 12 September 2025). The investigation was performed in strict accordance with the ethical principles of the Declaration of Helsinki. Patient confidentiality was maintained through rigorous data anonymization prior to analysis. Given the study’s retrospective nature, the institutional review board waived the requirement for informed consent.

### 2.2. Participant Selection and Definitions

The study cohort comprised 47 adult patients (≥18 years) diagnosed with ACPO and 50 age- and sex-matched healthy controls. Given the rarity of ACPO, all consecutive patients meeting the inclusion criteria during the study period were included to minimize selection bias. ACPO was defined based on clinical manifestations of acute large bowel obstruction—including abdominal distension, pain, and failure to pass flatus or stool—alongside radiological evidence of significant colonic dilatation without an identifiable mechanical blockage, as confirmed by computed tomography (CT) in all patients [1,5]. The control group consisted of 50 individuals undergoing routine health check-ups with normal complete blood counts and biochemical profiles, and without acute or chronic inflammatory conditions.

Patients were excluded if they presented with mechanical obstruction (e.g., volvulus, tumor, or stricture), active sepsis at admission, chronic autoimmune or inflammatory disorders, a history of malignancy under active treatment, or advanced hepatic or renal dysfunction. Additionally, patients with recent (<3 months) immunosuppressive or corticosteroid therapy and those with incomplete clinical, laboratory, or radiological records were excluded to ensure data integrity and minimize statistical bias. The sample consisted of all consecutive ACPO cases meeting the inclusion criteria during the study period.

The primary indications for surgical intervention were: (i) Clinical or radiological evidence of colonic ischemia or perforation (e.g., peritonitis, pneumoperitoneum), (ii) Failure of conservative and/or endoscopic management (e.g., persistent cecal dilation > 12 cm or clinical deterioration despite neostigmine or colonoscopic decompression), and (iii) Clinical deterioration despite appropriate conservative and/or endoscopic management.

### 2.3. Data Acquisition and Radiological Evaluation

Demographic and clinical variables—including age, sex, comorbidities, and etiologic triggers (postoperative, medical/systemic, pharmacologic/metabolic, trauma, or obstetric)—were systematically extracted from the Hospital Information Management System and archived patient files. Treatment modalities (conservative, neostigmine, endoscopic, or surgical [cecostomy/resection]) and clinical outcomes (hospital stay, complications, and mortality) were recorded. All measurements of maximum colonic diameter were performed digitally via the Picture Archiving and Communication System by independent radiologists blinded to clinical outcomes. All measurements of maximum colonic diameter used for statistical analyses were obtained exclusively from CT images at initial diagnosis. Plain abdominal radiography, when performed, was used only as a supportive imaging modality and for clinical monitoring, and was not utilized for quantitative diameter measurements or correlation analyses.

### 2.4. Laboratory Analysis and Inflammatory Indices

Venous blood samples were collected from all patients upon admission, prior to any therapeutic intervention. Laboratory parameters, including complete blood count (leukocytes, neutrophils, lymphocytes, monocytes, and platelets) and biochemical markers (C-reactive protein, albumin, and lactate), were analyzed using standardized automated laboratory analyzers. Based on the absolute peripheral blood counts, systemic inflammatory indices were derived using previously validated formulae [9]. Specifically, NLR was calculated as the absolute neutrophil count divided by the absolute lymphocyte count. The SII was defined as (platelets × neutrophils)/lymphocytes, while SIRI was determined using the formula (neutrophils × monocytes)/lymphocytes.

### 2.5. Statistical Analysis

Statistical analyses were conducted using SPSS version 27.0 (IBM Corp., Armonk, NY, USA). Data normality was assessed via the Shapiro–Wilk test. Continuous variables are expressed as median [interquartile range (IQR)] or mean ± standard deviation, as appropriate. Inter-group comparisons were performed using the Mann–Whitney U test or Kruskal–Wallis test. Spearman’s correlation coefficient was employed to evaluate associations between inflammatory indices and clinical parameters. The discriminatory performance of markers for surgical intervention was evaluated using receiver operating characteristic (ROC) curve analysis. Optimal cut-off values were determined by the Youden Index. AUC values were compared using the DeLong test to assess the discriminative superiority of SIRI, SII, and NLR. DeLong test comparisons were performed and verified using MedCalc version 23.4.8 (MedCalc Software Ltd., Ostend, Belgium). Composite inflammatory indices (SIRI, SII, NLR) were calculated retrospectively from archived laboratory data and were not available to the treating clinicians at the time of clinical decision-making, thereby minimizing incorporation bias. Univariate logistic regression was performed to identify candidate predictors (*p* < 0.10 or high clinical relevance), followed by a parsimonious multivariate model. To mitigate overfitting given the limited sample size (*n* = 47), the final model was restricted to a maximum of three independent variables. Model calibration was assessed via the Hosmer–Lemeshow test. A two-tailed *p* < 0.05 was considered statistically significant. Given the limited number of surgical events (*n* = 17), the statistical power for detecting differences between ROC curves and for multivariate modeling is acknowledged to be constrained; therefore, the results of this study should be interpreted as exploratory and hypothesis-generating.

## 3. Results

### 3.1. Baseline Characteristics and Clinical Etiology

The study included 97 participants: 47 patients with ACPO and 50 age- and sex-matched healthy controls. The mean age of the ACPO cohort was 69.2 ± 6.4 years, which did not differ significantly from that of the control group (68.3 ± 5.8 years; *p* = 0.482). Gender distribution was homogeneous between groups, with a male prevalence of 57.4% in the ACPO cohort and 52.0% in the control group (*p* = 0.778). The primary etiologic triggers identified for ACPO were medical/systemic disorders (*n* = 14, 29.8%), followed by pharmacologic or metabolic disturbances (*n* = 13, 27.7%), postoperative status (*n* = 10, 21.3%), trauma (*n* = 6, 12.8%), and obstetric causes (*n* = 4, 8.5%); detailed subcategories within each etiologic group are presented in Supplementary Table S1. Management was successful with conservative or endoscopic modalities in 63.8% (*n* = 30) of cases. Of the 30 patients managed non-operatively, 13 (27.7%) responded to conservative management alone, 10 (21.3%) required neostigmine, and 7 (14.9%) underwent endoscopic decompression. The failure of nonoperative management (*n* = 3) or evidence of ischemia/perforation (*n* = 14) necessitated surgical intervention in 17 cases. The selection of the procedure was exclusively determined by the intraoperative findings. To achieve definitive decompression, cecostomy was performed in eight patients with viable but refractory colonic distension, while colonic resection was performed for the nine patients with confirmed bowel necrosis or open perforation. This approach was adopted to reflect the actual clinical course and to avoid misclassification related to treatment crossover; sensitivity analyses excluding these crossover patients did not materially alter the observed between-group differences. All 47 ACPO patients (100%) underwent CT at initial diagnosis to confirm the diagnosis and exclude mechanical obstruction. Additionally, 28 patients (59.6%) also had plain abdominal radiography at the time of initial evaluation. The specific indications for surgical intervention among the 17 operated patients were: perforation/peritonitis (*n* = 6; 5 resections, 1 cecostomy), colonic ischemia/necrosis (*n* = 5; 4 resections, 1 cecostomy), and refractory colonic dilatation (>12 cm) despite conservative and/or endoscopic management (*n* = 6; all cecostomies). Colonic resection was performed when intraoperative findings demonstrated nonviable bowel, whereas cecostomy was the preferred decompressive approach for patients with refractory dilatation without evidence of bowel compromise.

The mean length of hospital stay for the entire ACPO cohort was 8.72 ± 4.68 days. Subgroup analysis demonstrated that patients requiring surgical intervention had a significantly longer hospital stay compared to those managed non-operatively (13.76 ± 2.96 vs. 5.87 ± 2.56 days; *p* < 0.001). Clinical complications were recorded in 8 patients (all within the surgical group, 17.0%), including surgical site infections (*n* = 4), wound dehiscence (*n* = 2), and pneumonia (*n* = 2). The overall in-hospital mortality rate was 10.6% (*n* = 5). All recorded fatalities occurred within the surgical subgroup, representing a 29.4% mortality rate for those requiring operative care. Of these, four deaths followed colonic resection, and one followed a cecostomy. No mortality was observed in patients successfully managed with conservative or endoscopic modalities.

### 3.2. Comparative Analysis of Hematologic and Inflammatory Indices

Comparative profiling revealed that systemic inflammatory activity was significantly higher in the ACPO group across all parameters (*p* < 0.001 for all). The SII in ACPO patients was nearly threefold higher than in controls (1436.0 vs. 552.9), and the SIRI was more than triple (3.06 vs. 0.86; *p* < 0.001). Similarly, WBC, CRP, and NLR values were significantly elevated in the ACPO cohort, while albumin levels were significantly lower. These findings are consistent with the presence of a significant systemic inflammatory state accompanying ACPO pathophysiology (Table 1).

### 3.3. Correlation with Disease Severity and Management Modality

**Figure 1:**
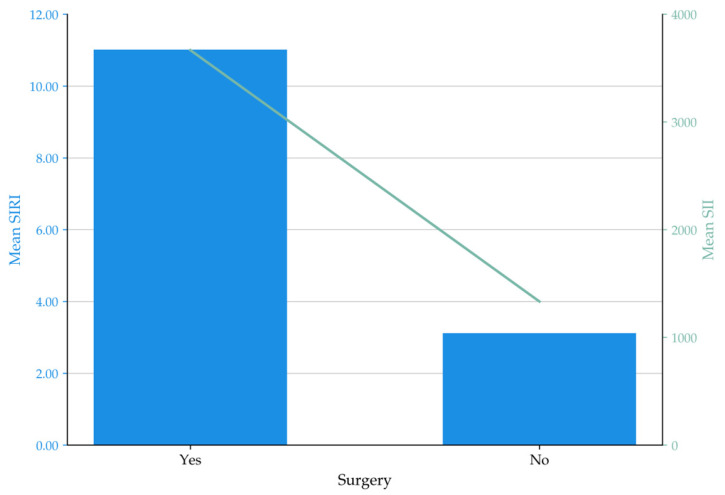
Comparison of mean Systemic Inflammation Response Index (SIRI) and mean Systemic Immune-Inflammation Index (SII) levels according to management modality (surgical vs. non-surgical). Bars represent mean SIRI values (left y-axis), while the green line represents mean SII values (right y-axis).

Within-cohort analysis revealed that inflammatory indices differed significantly between ACPO patients and healthy controls, and between surgically and non-operatively managed subgroups. Patients were categorized into two groups based on treatment outcome: those successfully managed non-operatively (conservative, neostigmine, or endoscopic; *n* = 30, median SIRI 2.3) versus those requiring surgical intervention (*n* = 17, median SIRI 11.2; *p* < 0.001). A similar stepwise escalation was observed for SII (1244.0 to 3374.0; *p* < 0.001) and NLR. Spearman’s correlation analysis confirmed significant positive associations between SIRI and both maximum colonic diameter (*r* = 0.69, *p* < 0.001) and length of hospital stay (*r* = 0.71, *p* < 0.001). Both SII and NLR correlated strongly with these clinical indicators (*r* = 0.61–0.68, *p* < 0.001). These findings demonstrate strong within-cohort correlations between inflammatory indices and measures of colonic diameter and hospital stay. The distribution of these indices across management modalities is illustrated in Figure 1.

### 3.4. Discriminatory Performance and Exploratory Modelling

**Figure 2:**
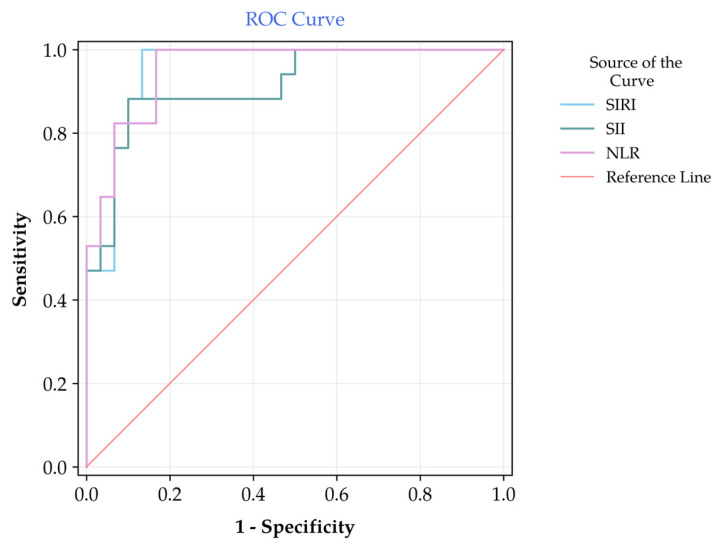
Receiver Operating Characteristic (ROC) curves of Systemic Inflammation Response Index (SIRI), Systemic Immune-Inflammation Index (SII), and Neutrophil-to-Lymphocyte Ratio (NLR) for predicting surgical intervention in patients with Acute Colonic Pseudo-obstruction.

ROC curve analysis demonstrated that hematologic indices showed within-cohort discriminatory performance for surgical intervention (Table 2, Figure 2). NLR and SIRI achieved the strongest performance, yielding an AUC of 0.840 (95% CI: 0.722–0.958) and 0.835 (95% CI: 0.718–0.952), respectively (*p* < 0.001). The discriminative superiority between these markers was further evaluated using the DeLong test, which revealed no statistically significant differences between the AUC of SIRI and SII (*p* = 0.242) or between SIRI and NLR (*p* = 0.911).

In the final multivariate logistic regression model, SIRI > 3.52 (adjusted OR: 8.2, 95% CI: 1.8–37.4, *p* = 0.007) and colonic diameter > 12 cm (adjusted OR: 6.4, 95% CI: 1.4–29.2, *p* = 0.016) were independently associated with surgical intervention within this cohort. The model demonstrated good calibration (Hosmer–Lemeshow, *p* = 0.645) and achieved a within-cohort classification accuracy of 88.9%, with sensitivity of 82.4% and specificity of 90.0% for surgical intervention (Nagelkerke R^2^ = 0.721). These within-sample performance estimates are descriptive and likely inflated in the absence of internal or external validation.

## 4. Discussion

This exploratory study provides preliminary evidence of a potential association between composite hematologic inflammatory indices—specifically SIRI, NLR, and SII—and the clinical progression and severity of ACPO. The data demonstrate that these indices are significantly elevated in ACPO patients compared with healthy controls and show within-cohort differentiation with respect to surgical intervention, with AUC values of 0.835 for SIRI and 0.840 for NLR. These findings indicate that such indices may reflect systemic inflammatory burden associated with intestinal compromise in Ogilvie’s syndrome, beyond their role as markers of systemic stress.

The demographic profile of the cohort, marked by a predominance of medical or systemic triggers and pharmacologic or metabolic disturbances, aligns with the global literature, which identifies ACPO as a condition primarily affecting elderly, multimorbid populations [1,2,3,4,5]. The identification of post-cesarean cases (8.5%) supports previous findings by Christensen et al., emphasizing that gastrointestinal symptoms in obstetric settings present unique diagnostic challenges [6,17]. While ACPO pathogenesis is traditionally attributed to autonomic imbalance involving parasympathetic deficiency or sympathetic overactivity [3,5,6], these results suggest that systemic inflammation may serve as a significant biological amplifier of neural disruption.

### 4.1. Mechanistic Considerations and Physiological Relevance

The observed association between these indices and ACPO severity may reflect cytokine-mediated impairment of enteric neuronal signaling. Pro-inflammatory mediators such as interleukin-6 and tumor necrosis factor-α can compromise smooth muscle contractility through nitric oxide–mediated pathways, potentially contributing to colonic atony and establishing a feedback loop wherein progressive distension promotes further inflammatory cell recruitment and ischemic injury [18]. The physiological relevance of SIRI, in particular, lies in its integration of monocyte counts alongside neutrophilia and lymphopenia [9,15,16]. Based on findings from other ischemic conditions [19], we hypothesize that prolonged colonic distension may impair intramural perfusion, and that monocyte dynamics—which may differ temporally from the early neutrophil response—could capture distinct aspects of the inflammatory cascade relevant to intestinal compromise. However, these mechanistic interpretations remain speculative, as direct tissue-level cytokine measurements and serial inflammatory profiling were not performed in this study.

### 4.2. Comparative Performance and Clinical Context

A key finding of this study is the within-cohort discriminatory performance of these markers. The DeLong test was used to compare the AUCs of SIRI, NLR, and SII, revealing no statistically significant differences (SIRI vs. SII, *p* = 0.242; SIRI vs. NLR, *p* = 0.911), indicating comparable overall accuracy. SIRI differs theoretically by integrating monocyte dynamics, whereas SII primarily reflects hemostatic activation via platelets. The multivariate analysis utilized a parsimonious model to minimize overfitting, incorporating the most significant variables and yielding 88.9% classification accuracy for surgical intervention. The consistent elevation of SIRI and NLR in both the ACPO-versus-control and the surgical-versus-conservative comparisons suggests consistent associations across clinical contexts. While CT imaging provides anatomical assessment of colonic distension, these inflammatory indices may provide complementary information reflecting systemic inflammatory status, suggesting that these indices may capture broader aspects of the systemic inflammatory response accompanying disease progression; however, their role in clinical decision-making remains unproven and requires prospective validation.

### 4.3. Integration with Established Literature on Gastrointestinal Emergencies

The utility of these inflammatory indices in ACPO aligns with their established performance in other acute gastrointestinal surgical conditions. Recent studies have demonstrated the predictive value of NLR, SII, and SIRI across various emergency surgical pathologies. Vural et al. [12] demonstrated that NLR, SII, SIRI, and PIV possessed acceptable diagnostic power (AUC: 0.738–0.765) in detecting strangulation in incarcerated inguinal hernias, with cut-off values providing sensitivities ranging from 62.6% to 76.9%. Similarly, Taşcı [13] reported that NLR played a pivotal role in predicting disease progression in benign intestinal obstructions, with high NLR values significantly increasing the risk of ischemia (22.66-fold), perforation (6.3-fold), post-operative complications (6.17-fold), and mortality (5.48-fold). The study identified an NLR cut-off of 6.56 for predicting ischemia with 82.9% sensitivity and 82.4% specificity, values remarkably consistent with our findings in ACPO.

Most recently, Ucaner et al. [14] investigated SII and PIV in patients with adhesive small bowel obstruction and found that these indices were significantly elevated in those requiring operative treatment (*p* = 0.010 and *p* = 0.015, respectively). While their ROC analyses yielded AUC values of 0.601 for SII and 0.596 for PIV—somewhat lower than our findings in ACPO—the study nonetheless supported the association between these markers and disease severity requiring operative management. The consistency of these findings across diverse gastrointestinal pathologies—from incarcerated hernias to adhesive obstructions—suggests that composite inflammatory indices may reflect a common pathophysiological mechanism involving intestinal compromise, ischemia, and systemic inflammatory response.

### 4.4. Clinical Implications and Radiological Integration

Historically, a colonic diameter exceeding 12 cm has been considered a critical threshold for perforation risk, with studies reporting that perforation rates may increase significantly beyond this limit [7,20]. Current management guidelines generally suggest conservative therapy for 48–72 h if the diameter remains below 12 cm and peritoneal signs are absent; however, reaching this threshold often triggers endoscopic decompression, neostigmine administration, or surgical consultation [1,3,7]. Our observations support this clinical context, demonstrated by the strong positive correlation between SIRI and maximum colonic diameter (*r* = 0.69, *p* < 0.001).

While colonic diameter is a cornerstone of radiological assessment, it may exhibit a temporal lag relative to the biological onset of transmural compromise [7,20]. Given that clinical symptoms can be masked in frail or sedated patients [8], exploratory threshold values such as SIRI > 3.52 and NLR > 4.22 may reflect underlying inflammatory processes in parallel with disease progression, occurring alongside the radiological evolution of colonic distension [2,7]. Such an integrated approach—combining radiological findings with biochemical markers—represents a hypothesis warranting prospective investigation, though the clinical decision-making value of these indices remains unproven.

### 4.5. Limitations and Future Directions

Several limitations merit consideration for interpreting these findings. First, the retrospective, single-center design and the relatively modest sample size (*n* = 47 ACPO patients, with only 17 surgical events) limit the generalizability of the findings and preclude a definitive temporal analysis. With only 17 surgical events, the events-per-variable ratio for our two-variable logistic regression model (approximately 8.5 EPV) falls below the commonly recommended threshold of 10 EPV, increasing the risk of model instability and overfitting. Second, while our multivariate model achieved a within-cohort classification accuracy of 88.9%, the lack of internal (bootstrap or cross-validation) and external validation cohorts means this value is likely inflated and should be considered a preliminary, within-sample estimate rather than generalizable performance. Third, we compared ACPO patients with healthy controls rather than disease controls (e.g., mechanical large bowel obstruction, toxic megacolon, postoperative ileus); while this confirms the presence of systemic inflammation in ACPO, it does not establish disease-specific diagnostic discrimination, and the absence of disease controls is a significant methodological limitation that precludes claims of diagnostic specificity. Fourth, the primary endpoint (surgical intervention) is a decision-dependent surrogate outcome influenced by clinician judgment, institutional protocols, and local risk-tolerance thresholds, rather than a purely objective pathophysiological endpoint such as histologically confirmed ischemia or necrosis. Importantly, the composite inflammatory indices evaluated in this study (NLR, SII, and SIRI) were not routinely reported or available to the treating clinicians at the time of clinical decision-making and were calculated retrospectively for study purposes only. Therefore, these indices did not directly influence surgical decision-making, although residual confounding related to overall disease severity cannot be fully excluded. Moreover, elevated inflammatory indices may simply reflect greater overall illness severity in surgically treated patients rather than independently predicting surgical necessity, representing potential confounding by indication. Because sicker patients are inherently more likely to undergo surgery and simultaneously exhibit higher inflammatory markers, the observed associations may be driven by disease severity itself rather than by a direct causal relationship between these indices and the need for operative intervention. Although the detailed surgical indication breakdown (perforation/peritonitis *n* = 6, ischemia/necrosis *n* = 5, refractory dilatation *n* = 6) partially mitigates this concern by demonstrating that the majority of surgical decisions were based on objective intraoperative findings, this limitation cannot be fully resolved without prospective studies incorporating predefined, protocol-driven surgical criteria. Fifth, blood samples were collected only at admission—serial monitoring during treatment could provide dynamic prognostic information, but was not performed in this retrospective analysis. Sixth, formal incremental value analyses (NRI, IDI, decision-curve analysis) were not performed to demonstrate whether composite indices provide additive prognostic information beyond classical markers such as CRP or WBC; these analyses were not feasible with only 17 patients. Seventh, although all patients underwent CT at initial diagnosis, 28 patients (59.6%) also had concurrent plain abdominal radiography at presentation, and serial colonic diameter monitoring during conservative management was performed using plain abdominal radiography, which may introduce measurement variability between diagnostic and follow-up assessments. The potential confounding influence of concurrent pharmacotherapy—particularly opioids and anticholinergics—on baseline inflammatory states was not independently stratified. Finally, our mechanistic interpretations of cytokines and monocyte recruitment remain hypothetical. Direct tissue-level or histological correlation was not available in this study. All cut-off values reported in this study are cohort-specific thresholds derived from Youden Index optimization and should not be applied in clinical practice without prospective external validation.

Future research should focus on prospective, multicenter validation studies with larger, more diverse patient populations. Serial measurements of inflammatory indices throughout the clinical course would provide valuable insights into their dynamic behavior and potential role in monitoring treatment response. Additionally, comparative studies including disease control groups (e.g., mechanical obstruction, toxic megacolon) would help establish the specificity of these biomarkers for ACPO. Finally, correlation with direct measurements of intestinal perfusion, tissue oxygenation, or cytokine profiles could elucidate the underlying pathophysiological mechanisms linking systemic inflammation to colonic compromise in ACPO.

## 5. Conclusions

In this exploratory, single-center study, composite inflammatory indices—particularly SIRI and NLR—were associated with disease severity and subsequent surgical intervention in patients with acute colonic pseudo-obstruction. A parsimonious model incorporating SIRI and colonic diameter demonstrated within-cohort discriminatory capacity; however, all performance estimates should be interpreted with caution given the limited sample size, absence of internal and external validation, and the decision-dependent nature of the surgical endpoint. Although these indices are derived from routine complete blood counts without additional cost, their clinical utility in ACPO remains unproven. The observed consistency between our findings and prior reports in other gastrointestinal emergencies is noteworthy but does not imply direct comparability across distinct pathophysiological entities. Prospective, multicenter studies incorporating disease control groups, serial biomarker assessment, and formal incremental value analyses are required before any clinical applicability can be considered.

## Figures and Tables

**Table 1:** Baseline Hematologic and Inflammatory Parameters.

Parameter	ACPO Group (*n* = 47)	Control Group (*n* = 50)	*p*-Value
White blood cells, ×10^9^/L	10.2 (8.0–12.5)	5.9 (5.0–7.0)	<0.001
C-reactive protein, mg/L	67.0 (40.0–108.0)	1.8 (1.7–2.0)	<0.001
Neutrophil-lymphocyte ratio	5.40 (3.5–10.0)	2.17 (2.1–2.2)	<0.001
SIRI	3.06 (1.9–7.4)	0.86 (0.8–0.9)	<0.001
SII	1436.0 (1022–2676)	552.9 (537–583)	<0.001
Albumin, g/dL	3.5 (3.1–3.8)	4.0 (3.8–4.2)	<0.001
Lactate, mmol/L	1.9 (1.5–2.5)	0.9 (0.8–1.0)	<0.001

Data presented as median (interquartile range). ACPO, acute colonic pseudo-obstruction; SIRI, systemic inflammation response index; SII, systemic immune-inflammation index. *p* < 0.05 was considered statistically significant.

**Table 2:** Within-Cohort Discriminatory Performance of Inflammatory Indices for Surgical Intervention.

Index	AUC (95% CI)	Cut-Off	Sensitivity (%)	Specificity (%)
NLR	0.840 (0.722–0.958)	>4.22	88.2	66.7
SIRI	0.835 (0.718–0.952)	>3.52	82.4	76.7
SII	0.795 (0.655–0.935)	>1203.5	82.4	63.3
CRP	0.812 (0.684–0.940)	>66.6	82.4	60.0
WBC	0.788 (0.648–0.928)	>10.1	82.4	63.3

AUC, area under the receiver operating characteristic curve; CI, confidence interval; CRP, C-reactive protein (mg/L); NLR, neutrophil-to-lymphocyte ratio; SII, systemic immune-inflammation index; SIRI, systemic inflammation response index; WBC, white blood cells (×10^9^/L).

## Data Availability

The data presented in this study are available on request from the corresponding author due to privacy and ethical restrictions.

## Supplementary Materials

The following supporting information can be downloaded at: https://www.mdpi.com/article/10.3390/diagnostics16040601/s1, Table S1: Detailed etiological triggers and specific inciting events for the ACPO cohort (*n* = 47).

## Abbreviations

The following abbreviations are used in this manuscript:

ACPO	Acute colonic pseudo-obstruction
SII	Systemic immune-inflammation index
SIRI	Systemic inflammation response index
NLR	Neutrophil-to-lymphocyte ratio
CRP	C-reactive protein
WBC	White blood cell
