# Supplementary material for: Systemic Inflammatory Indices and Disease Severity in Acute Colonic Pseudo-Obstruction: An Exploratory Retrospective Study
Source: Diagnostics (Basel). 2026 Feb 18;16(4):601. doi: 10.3390/diagnostics16040601 (PMC12939480; doi:10.3390/diagnostics16040601)
Supplement: Supplementary file 1 [file diagnostics-16-00601-s001.zip › diagnostics-4124836-supplementary.pdf]

Table S1. Detailed etiological triggers and specific inciting events for the ACPO cohort (n = 47).

Main Etiologic Category	Specific Inciting Events	*n*	%
**Medical/Systemic Disorders**		**14**	**29.8**
	Cardiorespiratory events (MI, CHF, COPD exacerbation, shock)	6	12.8
	Neurological diseases (stroke, Parkinson's disease, dementia)	5	10.6
	Non-septic systemic infections (pneumonia, viral infections)	3	6.4
**Pharmacologic/Metabolic**		**13**	**27.7**
	Medications (opioids, antipsychotics, anticholinergics)	8	17.0
	Electrolyte imbalances (hypocalcemia, hypokalemia)	5	10.6
**Postoperative Status**		**10**	**21.3**
	Major orthopedic surgery (hip or knee replacement)	6	12.8
	Spine surgery or other non-abdominal procedures	4	8.5
**Trauma**		**6**	**12.8**
	Thoracic or pelvic fractures (non-surgical)	4	8.5
	Spinal cord injury or major blunt trauma	2	4.3
**Obstetric Causes**		**4**	**8.5**
	Cesarean section	3	6.4
	Postpartum complications	1	2.1
**Total**		**47**	**100.0**

ACPO, acute colonic pseudo-obstruction; CHF, congestive heart failure; COPD, chronic obstructive pulmonary disease; MI, myocardial infarction. Subcategories are not mutually exclusive within each etiologic group. Percentages for subcategories are calculated relative to the total ACPO cohort (n = 47). The primary etiologic trigger was assigned based on clinical judgment when overlapping conditions were present.
